# Extraction, Purification, and Characterization of a Bacteriocin from Marine *Lactococcus lactis* NAN6399: Evaluating Antioxidant and Antimicrobial Activities

**DOI:** 10.3390/microorganisms14051030

**Published:** 2026-05-01

**Authors:** Fatma A. Ameen, Mahmoud E. Soliman, Amira M. Hamdan, Sherif F. Hammad

**Affiliations:** 1 Biotechnology, Basic and Applied Science Institute, Egypt-Japan University of Science and Technology, New Borg El Arab 21934, Egypt; 2 Botany and Microbiology Department, Faculty of Science, Alexandria University, Alexandria 21531, Egypt; 3 Faculty of Pharmacy, Ain Shams University, Cairo 11566, Egypt; mahmoud.soliman@ejust.edu.eg; 4 PharmD Program, Faculty of Pharmacy, Egypt-Japan University of Science and Technology (E-JUST), New Borg El Arab 21934, Egypt; sherif.hammad@ejust.edu.eg; 5 Oceanography Department, Faculty of Science, Alexandria University, Alexandria 21531, Egypt; amira_hamdan1978@yahoo.com; 6 Pharmaceutical Chemistry Department, Faculty of Pharmacy, Capital University (Formerly Helwan University), Cairo 11795, Egypt

**Keywords:** antimicrobial, antioxidant activity, bacteriocins, DPPH radical scavenging, lactic acid bacteria, *Lactococcus lactis*, Lactococcin972, marine microbiology, microbial biotechnology, proteomics

## Abstract

We evaluated the antimicrobial and antioxidant capabilities of a bacteriocin purified from a recently identified marine *Lactococcus lactis* (*L. lactis*) NAN6399 strain, a lactic acid bacterium recovered from Mediterranean coastal waters near Alexandria, Egypt, and identified by combined API 50 CHL phenotypic profiling and 16S rRNA gene sequencing. Bacteriocin purification was achieved by sequential ammonium sulfate precipitation and reverse-phase high-performance liquid chromatography (RP-HPLC). The purified bioactive fraction had an approximate molecular weight of 20 kDa by SDS-PAGE and a 106-amino-acid N-terminal sequence that, upon BLAST alignment, returned 98.1% overall identity to the Lactococcin 972 family bacteriocin AAK06118.1 from *L. lactis* IL1403, with divergence confined exclusively to the terminal two C-terminal residues. This sequence is structurally and functionally distinct from canonical Lcn972 (*L. lactis* IPLA 972): the two peptides share an identical 25-residue signal peptide but diverge entirely in their mature bioactive domains, which exhibit only 9.1% sequence identity. Canonical Lcn972 operates through Lipid II-mediated septum disruption and inhibits only *Lactococcus* species; the NAN6399 peptide, correctly designated as a novel member of the Lcn972-like peptide family, demonstrated broad-spectrum antimicrobial efficacy against multiple indicator organisms (*Staphylococcus aureus*, *Salmonella typhimurium*, *Escherichia coli*, *Klebsiella pneumoniae*, *Pseudomonas aeruginosa*, and *Enterococcus faecalis*), producing inhibition zones of up to 30 mm and minimum inhibitory concentration (MIC) values as low as 1.25 μg/mL against *S. aureus*. Antioxidant capacity was assessed using the DPPH radical scavenging assay, with the purified preparation achieving 73.14 ± 0.34% inhibition. Collectively, these data establish *L. lactis* NAN6399 as the producer of a bifunctional Lcn972-family bacteriocin with both antimicrobial and antioxidant potential, provide the first experimental characterization of the antimicrobial activity of this Lcn972-family branch, and highlight marine LAB as a productive reservoir for novel bioactive peptide discovery.

## 1. Introduction

Reactive oxygen species (ROS) serve as the principal catalysts of oxidative stress, a critical contributor to various pathological conditions, notably cancer. The detrimental effects of ROS manifest through their capacity to inflict harm on proteins, lipids, and nucleic acids, disrupting fundamental biochemical pathways and destabilizing the equilibrium necessary for normal cellular function [1]. Growing awareness of these mechanisms has catalyzed worldwide initiatives aimed at discovering natural compounds possessing robust antioxidant properties, which represent environmentally viable options compared with traditional therapeutic approaches.

The proliferation of pathogenic microorganisms exhibiting resistance to multiple antimicrobial drugs constitutes an escalating crisis for worldwide public health systems. Resistance strategies employed by microbes, such as enzymatic breakdown of antibiotics, active efflux transport systems, and biofilm formation, severely undermine the therapeutic effectiveness of currently available antimicrobial medications [2]. This situation highlights the critical necessity for discovering innovative antimicrobial compounds that operate through distinct mechanisms while producing fewer undesirable side effects.

Gram-positive microorganisms belonging to the lactic acid bacteria (LAB) taxonomic group have garnered considerable scientific attention owing to their capacity for synthesizing bacteriocins, ribosomally produced peptides that carry positive charges and exhibit powerful antimicrobial properties even at minimal quantities [3]. Bacteriocins produced by LAB are of particular importance because they are generally recognized as safe (GRAS) for human consumption, demonstrate selective antimicrobial action, and exhibit stability under diverse environmental conditions, making them attractive candidates for both food preservation and clinical applications [4,5]. The functional significance of LAB extends well beyond their antimicrobial capabilities to biotechnological applications in food production and preservation.

Oceanic ecosystems constitute an insufficiently explored source of microbial biodiversity and chemical novelty. Evolutionary pressures within competitive marine habitats have driven the development of unique metabolic pathways in marine microorganisms, rendering them valuable sources of bioactive compounds [6]. LAB originating from marine sources constitute a promising yet substantially underinvestigated research domain. Marine LAB bacteriocins may possess unique structural and functional characteristics distinct from those of terrestrial counterparts, potentially broadening the available spectrum of bioactive peptides. However, scientific investigations targeting marine-derived LAB and their bioactive metabolites remain scarce.

Egypt’s Mediterranean coastline, particularly the Alexandria region, constitutes an ecologically distinctive marine environment shaped by the convergence of multiple physicochemical and anthropogenic drivers. The proximity of the Nile Delta introduces substantial nutrient loads, organic matter, and freshwater discharge into the coastal waters, establishing marked salinity gradients and eutrophic conditions that structurally differentiate this environment from the characteristically oligotrophic open Mediterranean basin [7]. Superimposed upon these natural inputs are considerable anthropogenic pressures: Alexandria ranks among the most industrialized port cities on the southern Mediterranean coast, and its coastal waters receive continuous inputs from shipping activities, industrial effluents, and municipal discharge [8]. These combined stressors generate a high-competition, chemically complex microbial habitat in which resident microorganisms are subjected to intense selective pressure for the biosynthesis of bioactive secondary metabolites, including antimicrobial compounds, as adaptive competitive strategies [8,9]. The Alexandrian coastline has accordingly been identified as a productive ecological niche for the isolation of novel LAB strains with functionally distinct properties not commonly encountered in terrestrial or fermented food isolates [9]. These considerations collectively provide a robust ecological justification for prospecting this specific coastal environment as a source of bacteriocin-producing LAB with potentially novel structural and functional characteristics.

The present investigation aims to bridge the existing knowledge deficit through the isolation of a previously undescribed marine LAB strain, specifically *L. lactis* NAN6399, obtained from seawater samples gathered along this coastline. We describe the extraction, purification, and characterization of its bacteriocin product, a novel member of the Lcn972-like peptide family (AAK06118.1), designated herein as an Lcn972-family bacteriocin, with a molecular weight (MW) of approximately 20 kDa, and distinguish it from canonical Lcn972 at the primary sequence level. Additionally, antioxidant activity was assessed using DPPH radical scavenging methodology, and antimicrobial effectiveness was determined against a diverse panel of bacterial indicator organisms. Through the integration of molecular characterization with bioactivity evaluations, this research advances understanding of marine-sourced LAB and underscores their potential for yielding bioactive molecules applicable in both biomedical and industrial contexts.

## 2. Materials and Methods

### 2.1. Collection of Samples and LAB Isolation Procedure

**Figure 1:**
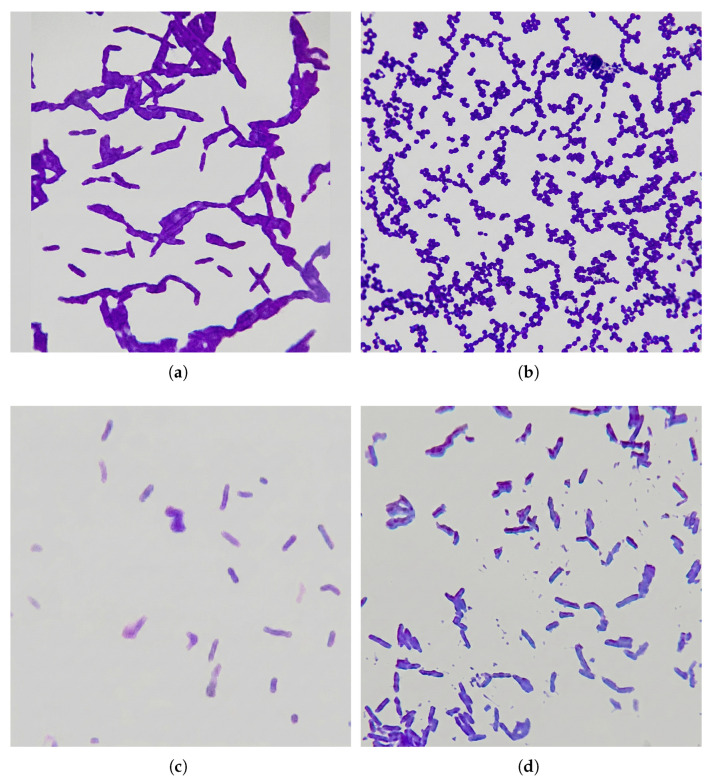
Microscopic characterization of selected bacterial isolates. Gram staining reveals the cellular structure and arrangement of the cells, confirming their Gram-positive status. Images were captured using an oil immersion objective (1000×). (**a**) Gram-stained micrographs of the isolate Lab 4, showing rod-shaped morphotypes; (**b**) Gram-stained micrograph of LAB isolate 1 (*L. lactis* NAN6399), showing cluster-forming cocci; (**c**) Gram-stained micrograph of LAB isolate 2, showing rod-shaped morphotypes; (**d**) Gram-stained micrograph of LAB isolate 5, showing rod-shaped morphotypes.

Seawater and sediment specimens totaling twenty were gathered from Egypt’s Mediterranean coastline in proximity to Alexandria, utilizing sterile Falcon tubes with 50 mL capacity under aseptic protocols, with subsequent storage maintained at 4 °C. Isolation of LAB from gathered specimens was accomplished using MRS media (Difco Laboratories, Detroit, MI, USA), employing spread plate procedure with incubation conducted at 30 °C over 48 h within anaerobic environments established using AnaeroPack sachets (Mitsubishi Gas Chemical Company, Inc., Tokyo, Japan) [10]. Colonies exhibiting catalase deficiency and showing Gram stain positive morphology were maintained using MRS agar slants at a temperature suitable for routine laboratory work. Those colonies were further characterized by examining their cellular morphology structure using a light microscopy under oil immersion (1000×) magnification where samples shows cluster-forming cocci as well as distinct rod shapes (Figure 1). Extended preservation was achieved by storing cultures in a glycerol solution at a concentration of 30% at temperature of −20 °C [9].

### 2.2. Laboratory Evaluation of Antimicrobial Properties in Selected LAB Strains

Cultivation of LAB isolates was initiated by introducing them into MRS broth media. Incubation extended over 48 h with temperature maintained at 30 °C, after which bacterial biomass was recovered via centrifugation applying an RCF of approximately 6000 over 0.25 h at 4 °C. Acellular supernatants were subsequently neutralized to neutral pH with 1 M sodium hydroxide solution. Sterilization of the supernatant was performed through filtration employing membranes with 0.45-micron pore sizes (Sartorius, Goettingen, Germany), with the resulting product designated as initial supernatant [11]. Preliminary qualitative screening of antimicrobial capabilities for the seven isolated LAB strains against selected pathogenic indicator organisms showed that the lab isolate LAB1 exhibited the highest antimicrobial activity against all tested bacterial strains (Table 1). Antimicrobial activity of the filtered supernatant was tested against a panel of six bacterial indicator strains (Table 2) using the agar well inhibition assay, as documented by [12]. Agar plates prepared using Mueller–Hinton medium formulation were inoculated utilizing overnight cultures at 1% concentration of target bacterial organisms. Following media solidification, wells measuring 4 mm in diameter were created using sterilized cork borers. Facilitation of extract diffusion was achieved by dispensing 100 μL volumes of crude solution into individual wells, with plates subsequently stored at 4 °C for 2 h. Subsequently, plates underwent incubation over 24 h at 37 °C under aerobic environmental conditions; measurement of bacterial inhibition zones surrounding individual wells was then performed.

### 2.3. Characterization Procedure for Selected LAB Isolate

The LAB variant obtained and demonstrating substantial antimicrobial capabilities underwent characterization utilizing the API carbohydrate fermentation kit (with the API 50 CHL medium) adhering to protocols specified by the manufacturer (BioMérieux, Lyon, France). Incubation was performed at a temperature of 30 °C. Fermentation profile observations were recorded following a duration of 24 h, and a second evaluation was conducted after 48 h of incubation. Results obtained from the fermentation profiles were interpreted using API WEB v5.0 database software [9].

Furthermore, the selected strain underwent sequence analysis of 16S ribosomal RNA conforming to methodologies detailed by [10]. Sequences produced during this investigation underwent comprehensive analysis and comparison against existing database entries using the BLAST algorithm, available online via the National Center for Biotechnology Information [13].

### 2.4. Bacteriocin Extraction and Recovery Methods

The culture procedure consisted of inoculating MRS media with the LAB variant, with incubation conducted over 24 h at 30 °C temperature. Cell isolation was accomplished through centrifugation utilizing identical centrifugation parameters previously described, with supernatant subsequently filter-sterilized and pH adjusted to 7.0. Precipitation of target components was achieved by incorporating ammonium sulfate at 70% concentration into supernatant fluid, followed by continuous stirring and storage at 4 °C extending over 24 h. Subsequent processing involved centrifugation at a temperature of 4 °C for a duration of 20 min using an RCF value of 8000 to recover the precipitate. The obtained precipitate underwent resuspension in potassium phosphate buffer at 0.1 M concentration (pH 7.2) and was subjected to dialysis utilizing identical buffer over 12 h at a temperature of 4 °C using dialysis tubing composed of cellulose polymer with acetate material characteristics, with a molecular weight exclusion limit of 1000 Daltons (Sigma-Aldrich, Darmstadt, Germany) (Figure S1). Following dialysis completion, the bacteriocin preparation underwent lyophilization and was preserved for subsequent purification procedure [11].

Chromatographic fractions obtained were subsequently assessed for inhibitory potential via the spot-on-lawn methodology as described by [14]. Assessment procedure involved application of 10 microliter aliquots from individual chromatographic fractions on agar plates seeded with *Klebsiella pneumoniae* ATCC 13883, incubated at 30 °C for 24 h. Antimicrobial activity was evaluated by measuring zone of inhibition around the application sites, quantified as arbitrary units per milliliter (AU/mL).

The lyophilized protein preparation underwent refinement using reverse-phase high-performance liquid chromatography (RP-HPLC). The instrument system employed was a model 1200 unit manufactured by Agilent Technologies, Inc. (Santa Clara, CA, USA), equipped with a C18 stationary phase column. Methodology conformed to protocols documented by [15]. Fractions that eluted were subsequently collected underwent evaluation for antibacterial capabilities utilizing the spot-placement antimicrobial assay methodology outlined by [14]. Specifically, 10 μL volumes of individual fractions were applied onto Mueller–Hinton culture medium plates previously prepared with 1% inoculum of *Klebsiella pneumoniae* ATCC 13883 and maintained at 30 °C over 24 h. Formation of inhibition zones surrounding individual application sites was monitored and reported as arbitrary unit (AU) designations per milliliter (AU/mL) utilizing methodology from [12]:

(1) $$\mathrm{Arbitrary}\;\mathrm{unit}\;\left(\mathrm{AU}/\mathrm{mL}\right)=\frac{\mathrm{inhibition}\;\mathrm{zone}\;\mathrm{diameter}\;\left(\mathrm{mm}\right)}{\mathrm{sample}\;\mathrm{volume}\;\mathrm{loaded}}\times 1000$$

### 2.5. Determination of Bacteriocin Molecular Weight and Concentration

Throughout individual purification stages, bacteriocin concentration was determined employing Bradford’s methodology [16] utilizing bovine serum protein serving as the reference standard. Moreover, MW assessment of protein compounds was conducted through 15% sodium dodecyl sulfate–polyacrylamide gel electrophoresis (SDS-PAGE) [17]. Gel staining was performed using Coomassie staining dye (R-250 Brilliant Blue; supplier: Sigma-Aldrich), with the protein fraction’s MW determined relative to reference markers ranging between 10 and 170 kDa.

### 2.6. Analysis of Protein Amino Acid Sequence Composition

The identified protein band underwent careful excision from the SDS-PAGE gel with subsequent elution in phosphate-buffered saline (PBS) conforming to techniques documented by [18]. Amino acid sequence determination was accomplished utilizing Edman degradation methodology. N-terminal protein identification was performed via model 477A (Applied Biosystems, Foster City, CA, USA) integrated with model 120A detection module employing PTH chemistry [19]. Amino acid sequences obtained underwent comparison with GenBank database entries utilizing BLAST software [13] for bacteriocin identification purposes.

### 2.7. DPPH Radical Scavenging Antioxidant Activity Assessment

According to protocols from [20], DPPH solution at $6\times 10^{-5}$ M concentration was formulated in methanol. Methanol-based DPPH radical solution at 1 mL volume was mixed with 1 mL purified bacteriocin. Following complete mixing, the sample underwent incubation in darkness at room temperature over 30 min. Subsequently, absorbance measurement was conducted at 517 nm wavelength. Activity calculation was performed using the formula:

(2) $$\mathrm{Scavenging}\;\mathrm{activity}\;\%=\frac{A_{\mathrm{blank}}-A_{\mathrm{test}}}{A_{\mathrm{blank}}}\times 100\%$$

where $A_{\mathrm{blank}}$ represents untreated control absorbance, and $A_{\mathrm{test}}$ denotes treated sample absorbance.

## 3. Results

### 3.1. Identification of Marine LAB Strains Producing Bacteriocins

Among the twenty marine specimens initially collected, seven LAB strains were isolated. Preliminary qualitative screening was performed on these seven isolates to evaluate their antimicrobial capabilities against the designated panel of pathogenic indicator organisms (Table 1). The screening results demonstrated varying degrees of antagonistic activity among the isolates.

Notably, a single LAB isolate (designated LAB 1) was the only strain to demonstrate consistent, broad-spectrum antimicrobial capabilities against all six tested bacterial organisms (Table 2).

Consequently, LAB 1 was selected as the optimal candidate strain for further quantitative evaluation, bacteriocin production, and comprehensive molecular characterization. Subsequent quantitative analysis of LAB 1 using the agar well diffusion method and minimum inhibitory concentration (MIC) assays revealed pronounced inhibition zones, with the most significant activity observed against *S. aureus* and *S. typhimurium* at 30 mm diameter, corresponding to MIC values of 1.25 and 2.5 μg/mL, respectively (Table 2).

### 3.2. Molecular and Biochemical Identification of LAB Isolate

The chosen isolate, designated LAB 1, exhibited Gram-positive characteristics, displayed catalase-negative properties, and demonstrated non-spore-forming morphology. Utilization of the API 50 CHL system for sugar fermentation pattern analysis revealed 99.9% identity matches *L. lactis*. In this context, molecular characterization of the LAB 1 isolate was accomplished via 16S ribosomal RNA gene sequencing, revealing 100% sequence identity with *L. lactis* NAN6399 (GenBank accession number PP528436.1), as documented in the database repository [13].

### 3.3. Purification and Molecular Characterization of Bioactive Bacteriocin

**Figure 2:**
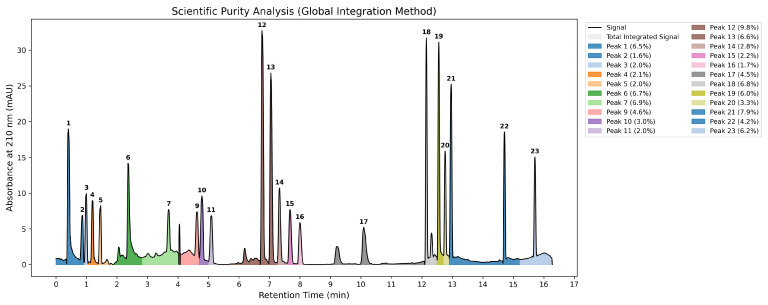
Computational purity analysis of the chromatographic profile. The global total area was integrated to calculate the relative abundance of each fraction. Colored regions represent distinct peaks detected by the valley-to-valley algorithm. This comprehensive integration method ensures that the purity percentage accounts for the entire signal, including baseline noise and minor impurities.

Crude protein extract obtained from NAN6399 underwent two-stage purification: initial precipitation employing ammonium sulfate followed by fractionation via RP-HPLC methodology. Quantitative analysis of the chromatographic peaks was performed using custom-developed computational algorithms. The raw chromatogram data was digitized and subjected to signal processing techniques to ensure data integrity. This included algorithmic smoothing using a Savitzky–Golay filter to enhance the signal-to-noise ratio and mathematical baseline correction to account for drift. Peak integration was automated using a valley-to-valley approach, calculating the area under the curve via the trapezoidal rule Figure 2. The reliability of this automated method was validated by comparing the calculated area of the major bioactive fraction against manual integration results.

**Figure 3:**
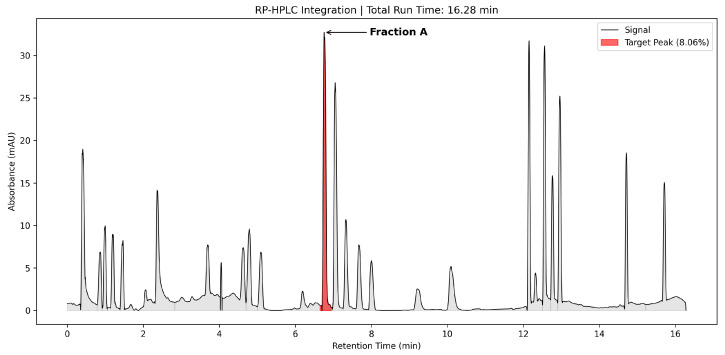
Reverse-phase high-performance liquid chromatography (RP-HPLC) profile of the bacteriocin extract. The arrow indicates the target bioactive fraction (Fraction A), which eluted at a retention time of approximately 6.78 min. The red shaded region highlights the integrated peak area. The total global area under the curve is shown in gray background.

**Figure 4:**
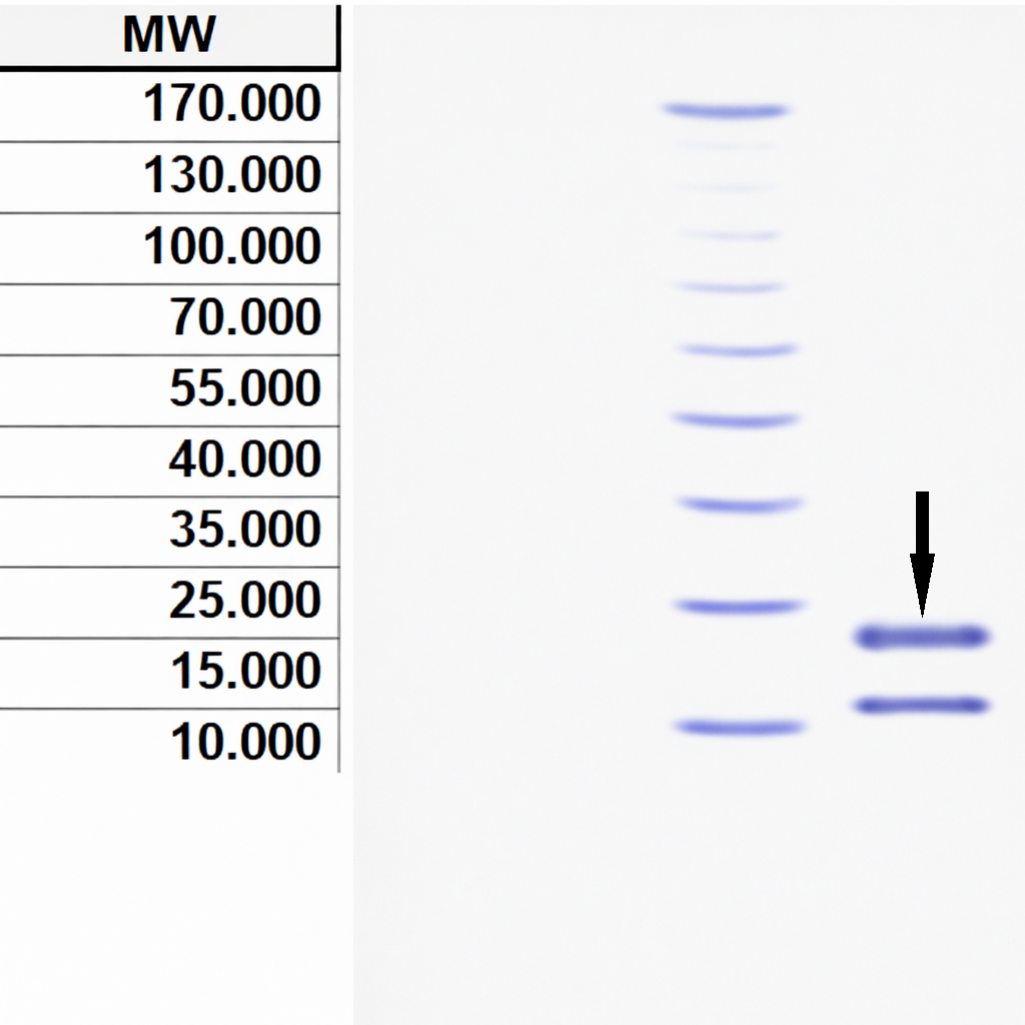
Electrophoretic profile demonstrates the molecular mass characteristics of the antimicrobial peptide generated by the chosen LAB strain. Track 1: molecular weight reference standards; Track 2: bacteriocin preparation obtained following purification from *L. lactis* NAN6399. The arrow indicates LAB 1 isolate with MW of 20 kDa.

Chromatographic analysis revealed a complex elution profile, with the most prominent peak (designated as Fraction A) eluting at a retention time of 6.78 min. Integration of the peak area indicated that this fraction constituted 8.14% of the total injected protein as shown in Figure 3, which coincided with the highest observed antimicrobial activity. Purified bacteriocin preparations displayed a singular band on SDS-PAGE analysis, with MW determination of approximately 20 kDa for bacteriocin synthesized by *L. lactis* when compared against respective protein MW standards presented in Figure 4. The bioactive bacteriocin band underwent N-terminal sequencing analysis using amino acid identification procedure. Protein sequencing yielded the following sequence information:

MQTKKLLVSTLILATLGGTLLQVSPVFAINRSTYSQGSTNDKKYGMGAYAAYWNNYGNHWAEVTYGDKYGGRVVSVHANQQAYAWLNTRWAEPATFYHSNGWVGVV

Sequence alignment studies implemented via BLAST platform algorithm against GenBank database entries identified the bacteriocin as Lcn972 with matching identity to NCBI reference sequence WP_010906269.1.

### 3.4. Radical Scavenging Antioxidant Capacity of Bacteriocin

Experimental results presented in Table 3 demonstrate that Lcn972 exhibited DPPH radical scavenging capacity achieving 73% reduction of the DPPH compound, determined using the previously described calculation formula.

## 4. Discussion

The isolation of only one bacteriocin-producing strain, *L. lactis* NAN6399, from twenty marine specimens reflects a selectivity pattern broadly consistent with the marine LAB literature. Muñoz-Atienza et al. [21] similarly reported that the majority of marine LAB isolates fail to produce bacteriocins with inhibitory activity against clinically relevant indicator organisms and attributed this low prevalence to the functional specificity of marine bacteriocin biosynthesis rather than to a true absence of antimicrobial capacity. The singular productive strain identified here (represented by LAB 1 among the seven LAB isolates recovered) nonetheless exhibited inhibitory activity against all six indicator organisms tested, a breadth of spectrum that substantially exceeds what is typically observed for a single LAB preparation and that represents the primary phenotypic finding warranting mechanistic investigation. In comparison with the remaining six LAB isolates, which displayed either no detectable activity or activity restricted to one or two indicator organisms, the combinatorial Gram-positive and Gram-negative coverage of NAN6399 is highly atypical for a *Lactococcus* strain, and this atypicality itself constitutes a scientifically significant observation [22].

The dual-method identification of the producing strain through API 50 CHL phenotypic profiling at 99.9% probability and 16S rRNA gene sequencing at 100% identity provides a high-confidence taxonomic assignment to *L. lactis* NAN6399 (GenBank PP528436.1) that is consistent with best-practice standards for LAB identification [23]. The convergence of an independent phenotypic and molecular result at near-perfect confidence values excludes the principal sources of misclassification that affect either method applied in isolation and establishes the producing strain’s identity with a degree of certainty sufficient to support the mechanistic and ecological inferences drawn from its antimicrobial profile.

Purification by sequential ammonium sulfate precipitation and RP-HPLC is a well-validated workflow for *L. lactis*-derived peptides, recently confirmed in the purification of nisin Z using an identical two-stage protocol [24]. In the present study, this approach yielded a complex elution profile in which the most prominent fraction (Fraction A, retention time 6.78 min) represented 8.14% of the total injected protein and coincided with the highest observed antimicrobial activity. SDS-PAGE of Fraction A revealed a single discrete band at approximately 20 kDa, a molecular mass considerably larger than the 3–10 kDa range characteristic of canonical class IIb bacteriocins [25], though comparable results have been reported for bacteriocins from *L. lactis* CH3 characterized under analogous conditions [26]. N-terminal sequencing of the band yielded a 106-amino-acid prepeptide sequence that, upon BLAST alignment, returned the highest-scoring match to the Lactococcin 972 family bacteriocin from *L. lactis* subsp. *lactis* IL1403 (AAK06118.1, UniProt Q9CE28). This identification requires precise contextualisation at the sequence level to accurately characterize the relationship between the peptide reported here, canonical Lcn972, and the AAK06118.1 reference entry—a distinction that is central to interpreting the biological findings of the study.

Canonical Lcn972, first characterized by Martínez et al. [27] from *L. lactis* IPLA 972, is a 91-amino-acid prepeptide comprising a 25-residue signal peptide followed by a 66-residue mature bacteriocin. Its complete prepeptide sequence is:

MQTKKLLVSTLILATLGGTLLQVSPEGTWQHGYGVSSAYSNYHHGSKTHSATVVNNNTGRQGKDTQRAGVWAKATVGRNLTEKASFYYNFW

where the first 25 residues (MQTKKLLVSTLILATLGGTLLQVSP) constitute the signal peptide and the remaining 66 residues constitute the mature bioactive domain. Canonical Lcn972 operates through a highly specific interaction with Lipid II at the cell division septum, inhibiting peptidoglycan incorporation without forming membrane pores, and this mechanism restricts its inhibitory spectrum exclusively to susceptible *Lactococcus* species [27,28].

The 106-amino-acid sequence determined by N-terminal sequencing in the present study is:

MQTKKLLVSTLILATLGGTLLQVSPVFAINRSTYSQGSTNDKKYGMGAYAAYWNNYGNHWAEVTYGDKYGGRVVSVHANQQAYAWLNTRWAEPATFYHSNGWVGVV

A direct comparison of the two sequences reveals that, while both share an identical 25-residue signal peptide (MQTKKLLVSTLILATLGGTLLQVSP), the mature bioactive domains are fundamentally different. The mature domain of canonical Lcn972 (66 residues, initiating at EGTW…) and the mature domain of the NAN6399 peptide (81 residues, initiating at VFAI…) share only 9.1% sequence identity over the alignable region, a level of divergence that unambiguously precludes functional equivalence. The overall prepeptide identity between canonical Lcn972 and the NAN6399 sequence is 34.1% over 91 aligned positions, with the apparent similarity inflated almost entirely by the shared signal peptide. By contrast, alignment of the NAN6399 sequence against the AAK06118.1 entry from *L. lactis* IL1403—a 108-amino-acid prepeptide encoding an 83-residue mature domain—reveals 98.1% overall identity across 106 aligned positions. The two sequences share an identical 104-residue prefix, diverging only at the ultimate four positions: AAK06118.1 terminates in TRSW, whereas the NAN6399 peptide terminates in VV, two residues shorter. The mature peptide identity between NAN6399 and AAK06118.1 is 97.5%, confirming that the NAN6399 peptide is a C-terminal variant of AAK06118.1 rather than a variant of canonical Lcn972. These three sequences therefore represent distinct members of the Lcn972-like peptide family (InterPro IPR006540): canonical Lcn972 constitutes the founding narrow-spectrum member, while AAK06118.1 and the NAN6399 peptide constitute a structurally related but functionally distinct branch, differentiated from canonical Lcn972 at the mature domain level by the complete replacement of the EGTW-initiating bioactive sequence with an independent VFAI-initiating domain of greater length. Crucially, AAK06118.1 has, to the authors’ knowledge, not been functionally characterized in terms of its antimicrobial spectrum, inhibitory potency, or mechanism of action. The present study therefore constitutes the first experimental characterization of the antimicrobial and antioxidant activities of this Lcn972-family branch, represented here by its NAN6399 C-terminal variant.

The broad-spectrum inhibitory activity documented in the present study (encompassing both Gram-positive and Gram-negative organisms) is accordingly not paradoxical in relation to the peptide’s correct sequence identity, as it would have been if canonical Lcn972 had been the actual product. Rather, the observed phenotype is consistent with a structurally distinct family member whose mature domain is unrelated to the Lipid II-binding sequence of canonical Lcn972 and which may, therefore, operate through an entirely different mechanism. The activity against *K. pneumoniae*, *P. aeruginosa*, and *E. coli*, all of which possess outer membrane permeability barriers that exclude many bacteriocins, nevertheless warrants mechanistic explanation. An expanded-spectrum variant of this family could operate through electrostatic membrane disruption, a well-characterized mode of action for cationic marine-derived antimicrobial peptides that targets the negatively charged phospholipid and lipopolysaccharide components of both Gram-positive and Gram-negative membranes [29,30]. This is not without LAB bacteriocin precedent: Wu et al. [31] demonstrated that bacteriocin ZFM216 exerts its effect through direct disruption of the transmembrane electrochemical potential, causing rapid collapse of the transmembrane potential difference (ΔΨ), depletion of intracellular ATP, and loss of membrane integrity without engaging the Lipid II pathway, establishing that membrane-targeting modes of action are available within the LAB bacteriocin structural repertoire and fully compatible with Gram-negative coverage. Marine selective pressures would provide additional ecological rationale for the evolution of broad-spectrum activity: marine microbial communities are dominated by Gram-negative proteobacteria entirely absent from the dairy and fermented food environments in which most characterized *L. lactis* strains have been isolated [32,33], and sustained competitive exposure to these organisms would plausibly drive the maintenance or amplification of Gram-negative inhibitory capacity in a marine-adapted producer.

It is additionally noted that the possibility of multi-bacteriocin co-production by *L. lactis* NAN6399 cannot be excluded on the basis of the current data. The capacity of individual LAB strains to simultaneously produce multiple structurally distinct bacteriocins with complementary spectra is well-documented [34,35], and the complex RP-HPLC elution profile observed beyond Fraction A provides circumstantial support for this interpretation. The antimicrobial activity of fractions beyond Fraction A was not independently assessed, constituting a meaningful limitation of the current work. Complete characterization of the bacteriocinogenic capacity of NAN6399 should accordingly include whole-genome sequencing with bioinformatic annotation of all bacteriocin biosynthetic gene clusters, independent antimicrobial activity testing of all resolved RP-HPLC fractions, and LC-MS/MS peptidome analysis of the culture supernatant, approaches increasingly recognized as essential for capturing the full antimicrobial potential of LAB strains [36].

The quantitative antimicrobial data reinforce the significance of the bioactive fraction. The inhibition zone of 30 mm recorded against *S. aureus* substantially exceeds values reported in comparable studies employing ammonium sulfate-purified bacteriocin preparations from enterococcal and lactic acid bacteria sources: Erginkaya et al. [37] reported inhibition zones of 5–13 mm against *S. aureus*, *E. coli*, and *S. Enteritidis* for partially purified enterocins from *E. faecium* and *E. faecalis*, while Ünal Turhan et al. [38] reported inhibition zone diameters of 8.66–9.25 mm for partially purified enterocins from the same genera against *L. monocytogenes* under identical disk diffusion conditions. The 30 mm value obtained in the present study is therefore markedly superior across comparable methodological conditions and provides objective evidence of the elevated potency of the NAN6399 preparation. MIC values of 1.25 and 2.5 μg/mL against *S. aureus* and *S. typhimurium*, respectively, are among the most potent values reported in the recent LAB bacteriocin literature for these organisms; Choi et al. [39] characterised bacteriocins from *L. lactis* and *Pediococcus pentosaceus* against the same species, reporting MIC values in the range of 2.5–20 μg/mL, placing the lower bound of their data in alignment with the most favourable values obtained in the present study. The comparatively elevated MIC of 20 μg/mL recorded against *E. faecalis* is consistent with the well-documented reduced susceptibility of *Staphylococcus* and *Enterococcus* species to LAB-derived bacteriocins, attributable in part to the capacity of these organisms to elaborate viscous polysaccharide capsules that impede the penetration of antimicrobial compounds into the bacterial cell [37]. Activity against *S. aureus* at 1.25 μg/mL is of particular clinical relevance given that this organism (and its methicillin-resistant form (MRSA)) remains one of the highest-priority targets on the WHO list of bacteria requiring novel antimicrobial strategies [25]. The broad-spectrum BLIS-producing LAB characterized by Thuy et al. [40] against overlapping pathogen panels, including *E. coli*, *K. pneumoniae*, and *P. aeruginosa*, further corroborates the plausibility of such a profile in a LAB-derived fraction, while the activity against these inherently Gram-negative organisms with outer membrane-mediated resistance directly reinforces the mechanistic hypotheses advanced above.

Beyond its antimicrobial properties, the purified Lcn972-family bacteriocin preparation demonstrated substantial DPPH radical scavenging activity, achieving a mean inhibition of 73.14 ± 0.34%. This value positions the bioactive fraction in the upper tier of antioxidant capacity documented for LAB-derived preparations in the contemporary literature. Hu et al. [41] reported DPPH scavenging rates ranging from 28.81 to 82.75% across LAB strains with strong antioxidant function, classifying strains above 70% as strongly antioxidant, a threshold comfortably exceeded by the preparation reported here. In contrast, Hamdaoui et al. [42] reported scavenging capacities of 10.56–26.29% for intact *L. lactis* cells, substantially lower values that likely reflect the difference between whole-cell.

Collectively, the findings of this study establish *L. lactis* NAN6399 as a marine-adapted producer of a novel Lcn972-family bacteriocin that is demonstrably distinct from canonical Lcn972 at the primary sequence level, sharing only a conserved signal peptide while diverging entirely in its mature bioactive domain. The work also demonstrates the inadequacy of BLAST-based annotation alone as a functional identifier for novel bacteriocins and advocates for the integration of genome-guided and mass spectrometry-based discovery approaches to fully resolve the bacteriocinogenic capacity of this strain. The marine environment, long underexplored as a reservoir for LAB-derived antimicrobials, emerges from this study as a productive ecological niche for the discovery of bifunctional bacteriocin-like peptides with expanded inhibitory spectra, a finding with substantive implications for the pipeline of next-generation antimicrobial agents against WHO-priority pathogens, and one that warrants systematic prospecting of marine LAB diversity as a strategic priority in the post-antibiotic era.

## 5. Conclusions

*Lactococcus lactis* NAN6399, isolated from Mediterranean coastal waters near Alexandria, Egypt, was confirmed as the producer of a bifunctional bacteriocin belonging to the Lcn972-like peptide family, exhibiting potent broad-spectrum antimicrobial activity and substantial antioxidant capacity in a single purified preparation. The purified fraction demonstrated MIC values as low as 1.25 μg/mL against *S. aureus* and 2.5 μg/mL against *S. typhimurium*, inhibition zones of up to 30 mm against the full panel of tested pathogens, and a DPPH radical scavenging inhibition of 73.14 ± 0.34%, placing it in the upper tier of both antimicrobial and antioxidant capacity documented in the contemporary LAB bacteriocin literature.

Sequence analysis established that the 106-amino-acid NAN6399 peptide is a C-terminal variant of the previously uncharacterized Lactococcin 972 family bacteriocin AAK06118.1 from *L. lactis* IL1403, sharing 98.1% overall identity, and is fundamentally distinct from canonical Lcn972: the two peptides share only a conserved 25-residue signal peptide, while their mature bioactive domains share just 9.1% sequence identity. This study therefore provides the first experimental characterization of antimicrobial and antioxidant activities for this Lcn972-family branch. The broad-spectrum activity against both Gram-positive and Gram-negative pathogens is consistent with a mechanism independent of the Lipid II-binding pathway that restricts canonical Lcn972 to *Lactococcus*-only inhibition, and its mechanistic basis warrants resolution through membrane permeability assays, transmembrane potential measurements, Lipid II binding competition experiments, whole-genome sequencing, and LC-MS/MS peptidome analysis of the producing strain.

These findings establish marine-derived *L. lactis* as a productive and underexplored source of bifunctional bacteriocin-like peptides with expanded inhibitory spectra, and position *L. lactis* NAN6399 as a promising biological scaffold for the development of next-generation antimicrobial and antioxidant agents. Systematic prospecting of marine LAB diversity represents a strategically valuable priority in the ongoing response to the global antimicrobial resistance crisis, with potential applications spanning clinical therapeutics, functional food preservation, and nutraceutical formulation.

## Figures and Tables

**Table 1:** Preliminary qualitative screening of antimicrobial capabilities for the seven isolated LAB strains against selected pathogenic indicator organisms. Presence (+) or absence (−) of antimicrobial activity is indicated.

LAB Isolate	*S. aureus*	*E. faecalis*	*K. pneumoniae*	*P. aeruginosa*	*E. coli*	*S. typhimurium*
LAB 1	+	+	+	+	+	+
LAB 2	−	−	+	+	−	+
LAB 3	−	−	−	−	−	−
LAB 4	−	−	−	−	−	+
LAB 5	−	−	−	+	−	+
LAB 6	−	−	−	−	−	−
LAB 7	−	−	−	+	−	−

**Table 2:** Evaluation of antimicrobial capabilities of LAB 1 isolate against selected bacterial organisms. Data represents mean zone of inhibition diameter (mm) ± standard deviation of three independent replicates (n=3) and minimum inhibitory concentration (MIC).

Pathogenic Organisms	Zone of Inhibition (mm)	MIC (μg/mL)
*E. faecalis*	18±1.0	20
*K. pneumoniae*	22±1.5	5
*E. coli*	20±1.2	10
*S. aureus*	30±0.6	1.25
*S. typhimurium*	30±1.2	2.5
*P. aeruginosa*	23±1.0	5

**Table 3:** Spectrophotometric Absorbance and DPPH Radical Reduction Capacity (%) of Lcn972 using mean control of diluted solution of DPPH of a mean measurement 0.226.

Bacteriocin Tested	Replicate	Spectrophotometric Reading (517 nm)	Control Reading (517 nm)	DPPH Inhibition (%)	Mean% Inhibition ± SD
Lcn972	1	0.0603	0.227	73.44	73.14 ± 0.34
	2	0.058	0.213	72.77	
	3	0.064	0.239	73.22	

## Data Availability

The original contributions presented in this study are included in the article and Supplementary Materials. Further inquiries can be directed to the corresponding author.

## Supplementary Materials

The following supporting information can be downloaded at: https://www.mdpi.com/article/10.3390/microorganisms14051030/s1, Figure S1: Dialysis procedure for the partial purification of the bacteriocin extract; Figure S2: Collection of marine seawater and sediment specimens using sterile 50 mL Falcon tubes. Figure S3: Representative images showing the antimicrobial activity of the marine LAB 1 isolate against test pathogens.

## Abbreviations

ABI	Applied Biosystems (Referring to the model of the protein sequencer)
API	Analytical Profile Index
ATCC	American Type Culture Collection
AU/mL	Arbitrary Unit per Milliliter
BLAST	Basic Local Alignment Search Tool
DMSO	Dimethyl Sulfoxide
DPPH	Diphenyl picrylhydrazyl
*E. coli*	*Escherichia coli* ATCC 25922
*E. faecalis*	*Enterococcus faecalis* JCM 5803
ELISA	Enzyme-Linked Immunosorbent Assay
JCM	Japan Collection of Microorganisms.
*K. pneumoniae*	*Klebsiella pneumoniae* ATCC 13883
*L. lactis*	*Lactococcus lactis*
LAB	Lactic Acid Bacteria
Lcn972	Lactococcin 972
MRS	de Man, Rogosa, and Sharpe (Agar Media)
MW	Molecular Weight
*P. aeruginosa*	*Pseudomonas aeruginosa* ATCC 27853
PBS	Phosphate-Buffered Saline
ROS	Reactive Oxygen Species
RP-HPLC	Reverse Phase High-Performance Liquid Chromatography
*S. aureus*	*Staphylococcus aureus* ATCC 25923
*S. typhimurium*	*Salmonella typhimurium* ATCC 14025
SDS-PAGE	Sodium Dodecyl Sulfate Polyacrylamide Gel Electrophoresis
