# Supplementary figures and images for: Extraction, Purification, and Characterization of a Bacteriocin from Marine Lactococcus lactis NAN6399: Evaluating Antioxidant and Antimicrobial Activities
Source: Microorganisms. 2026 May 1;14(5):1030. doi: 10.3390/microorganisms14051030 (PMC13210041; doi:10.3390/microorganisms14051030)

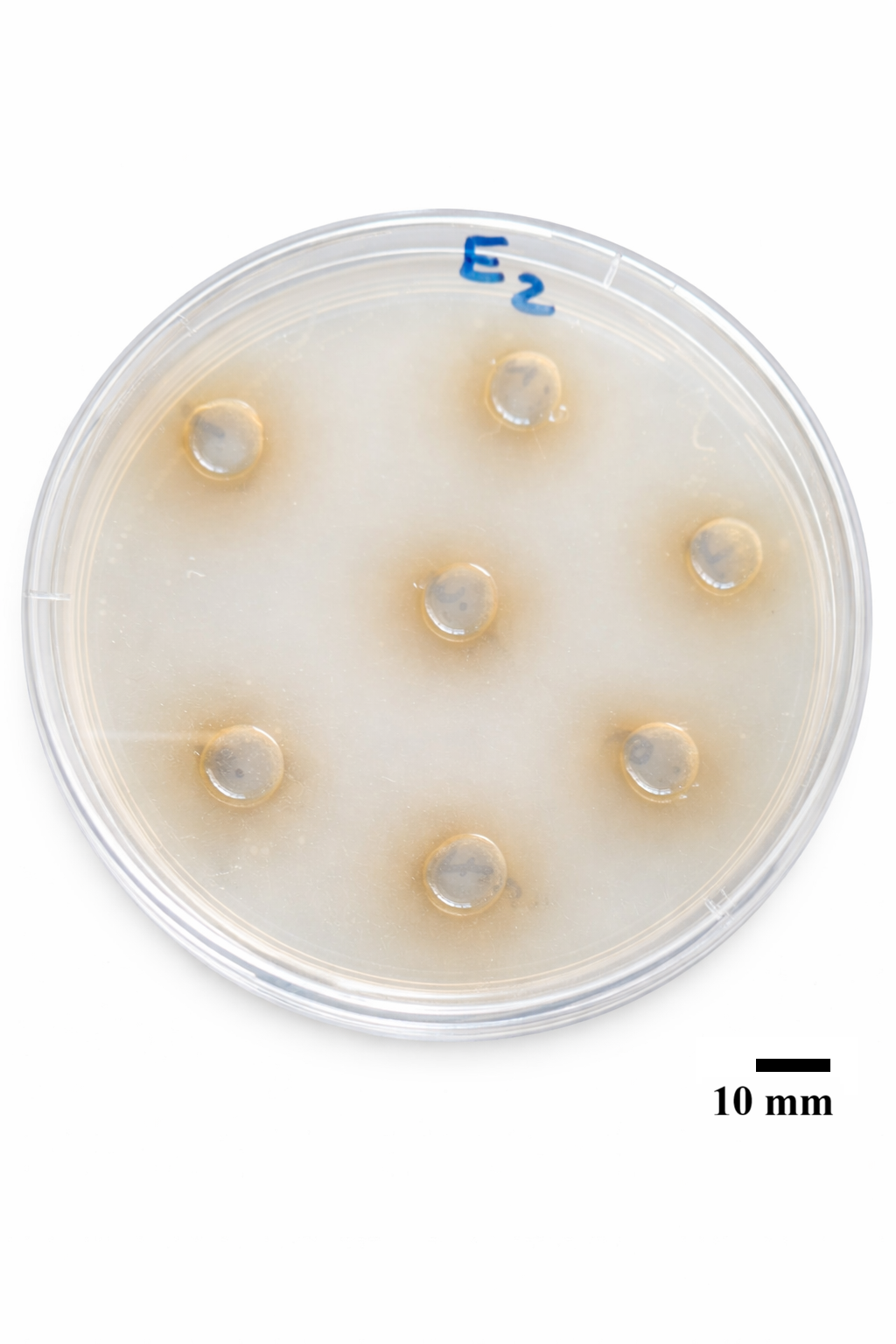

Supplement: Supplementary file 1 [file microorganisms-14-01030-s001.zip › Figure S3 - antimicrobial_EC.png]

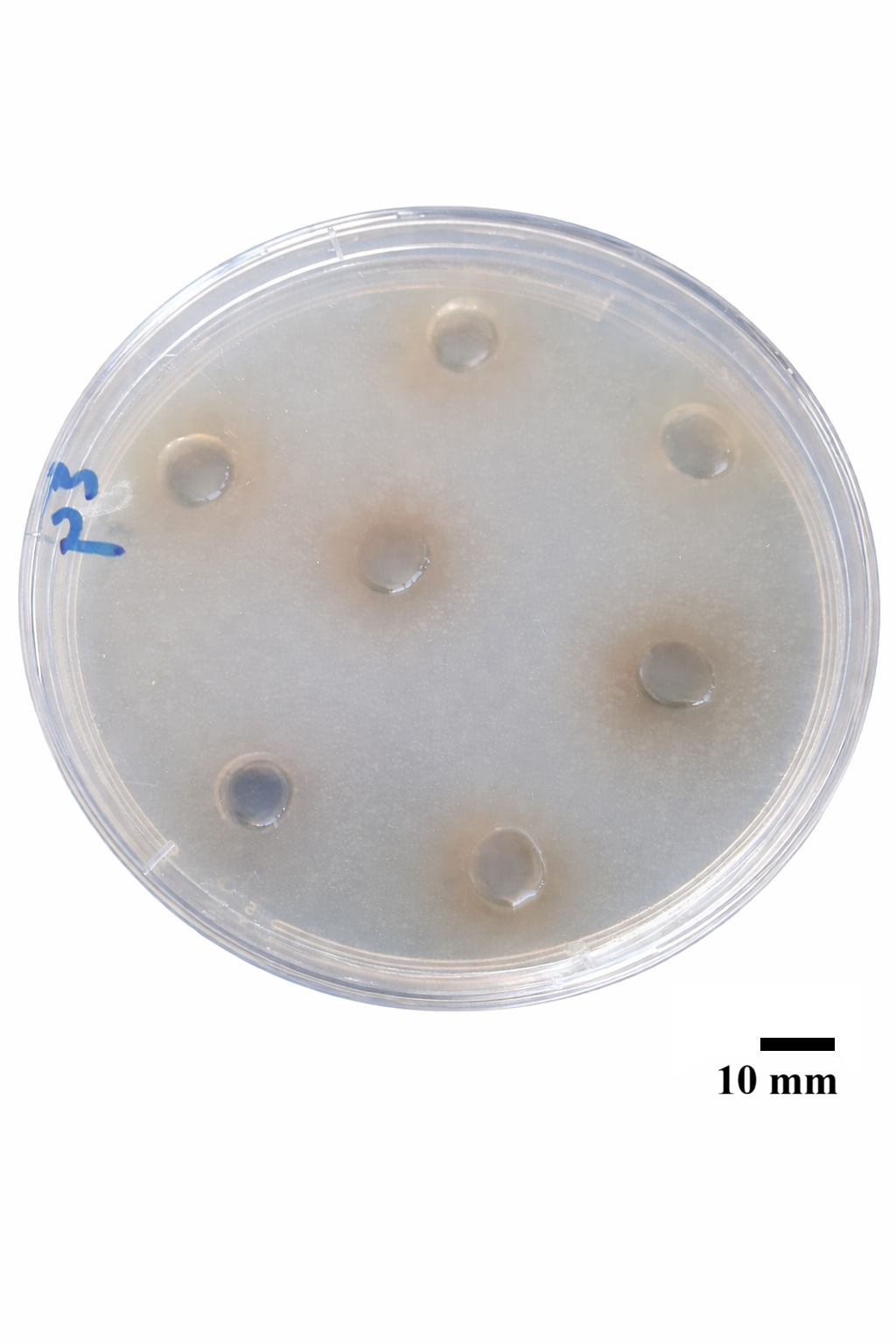

Supplement: Supplementary file 1 [file microorganisms-14-01030-s001.zip › Figure S3 - antimicrobial_EF.png]

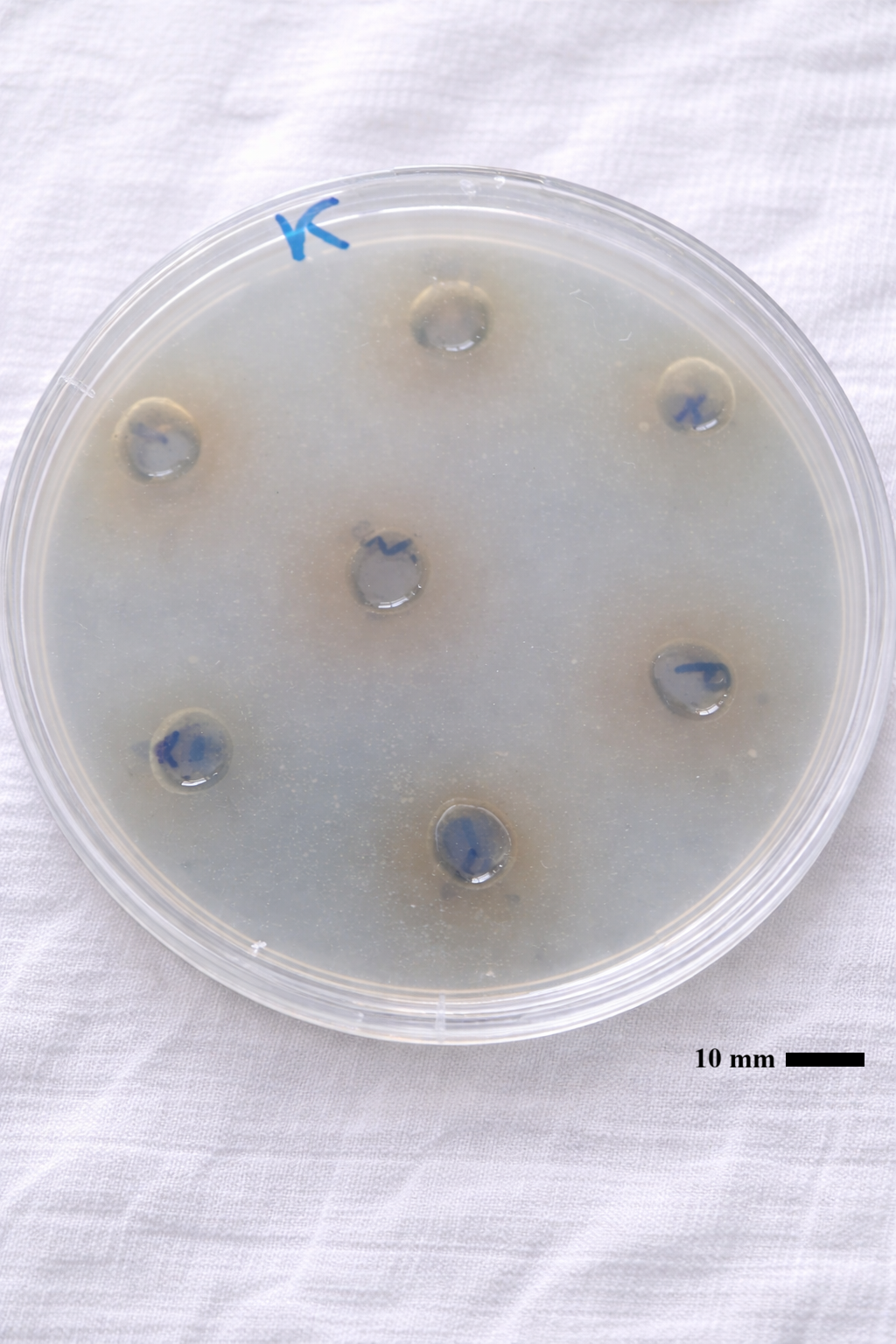

Supplement: Supplementary file 1 [file microorganisms-14-01030-s001.zip › Figure S3 - antimicrobial_KP.png]

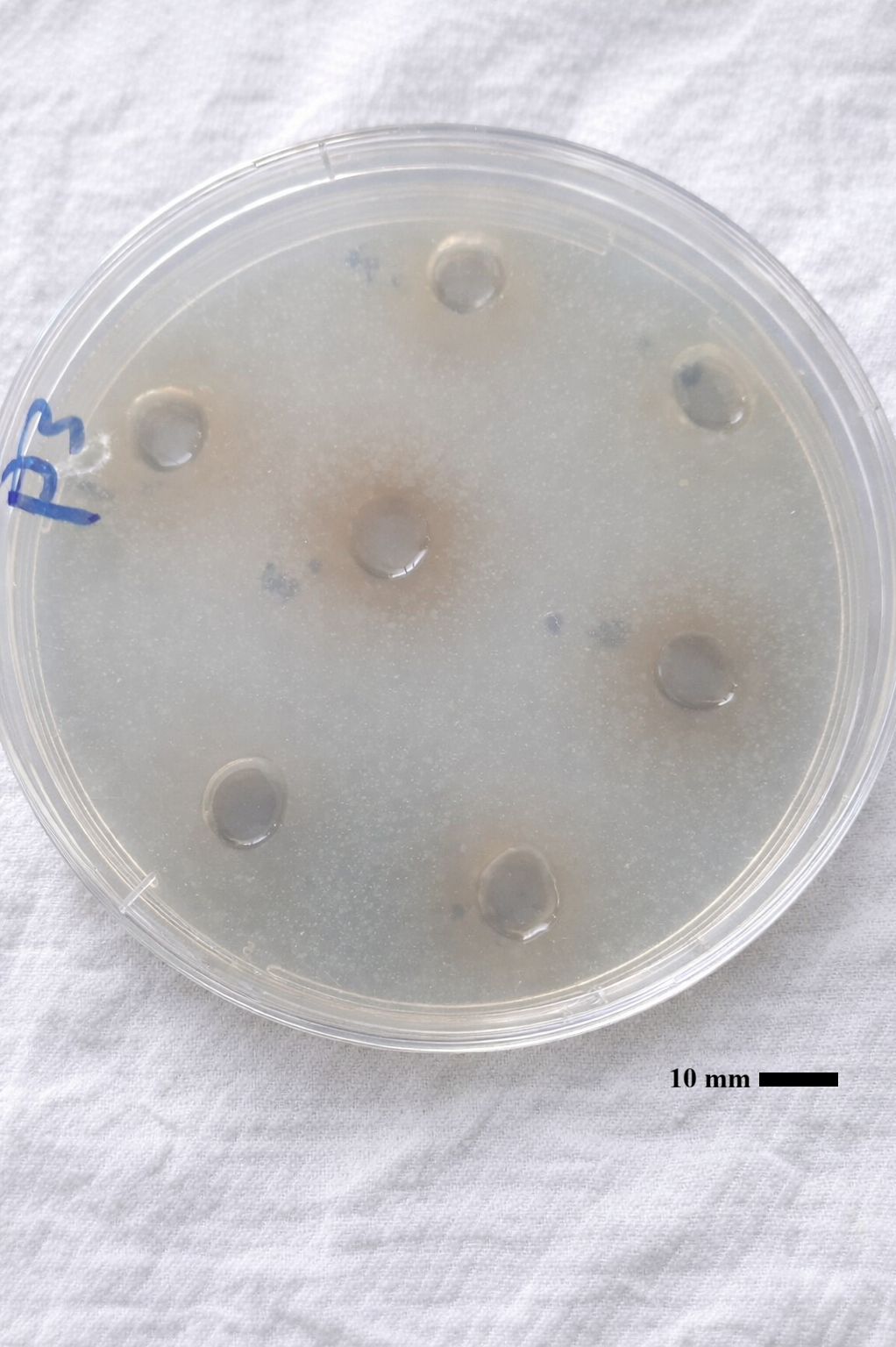

Supplement: Supplementary file 1 [file microorganisms-14-01030-s001.zip › Figure S3 - antimicrobial_PA.png]

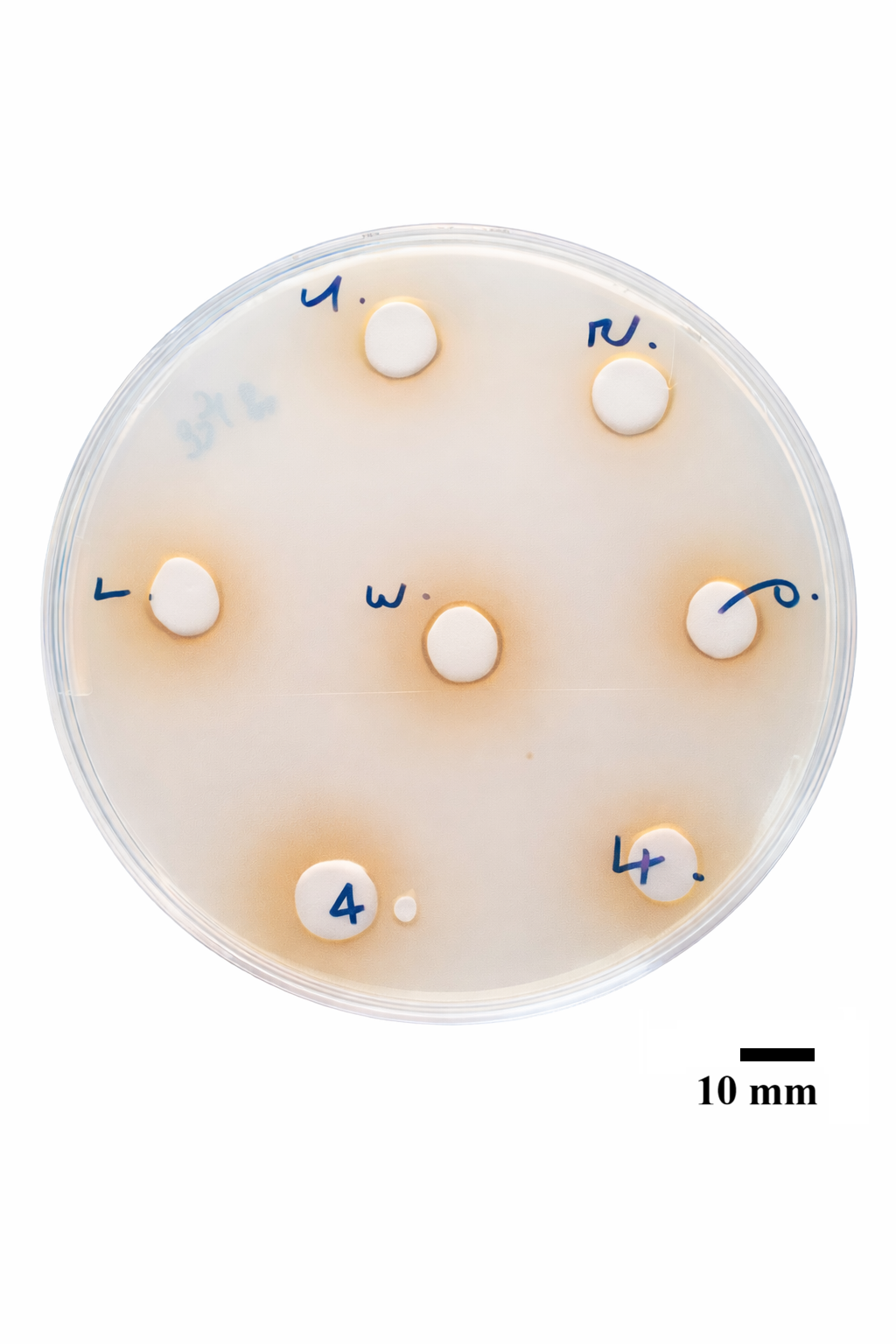

Supplement: Supplementary file 1 [file microorganisms-14-01030-s001.zip › Figure S3 - antimicrobial_SA.png]

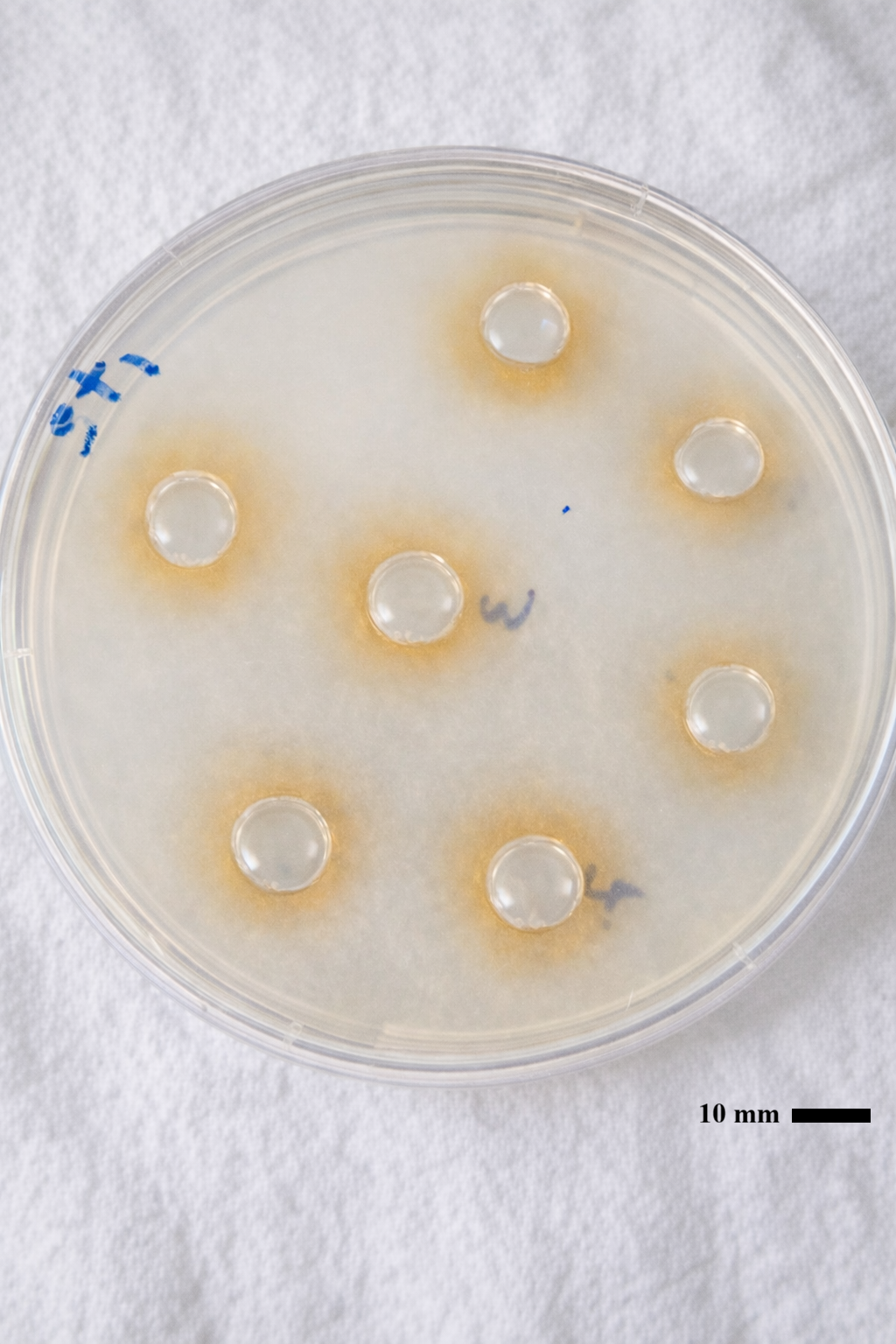

Supplement: Supplementary file 1 [file microorganisms-14-01030-s001.zip › Figure S3 - antimicrobial_ST.png]

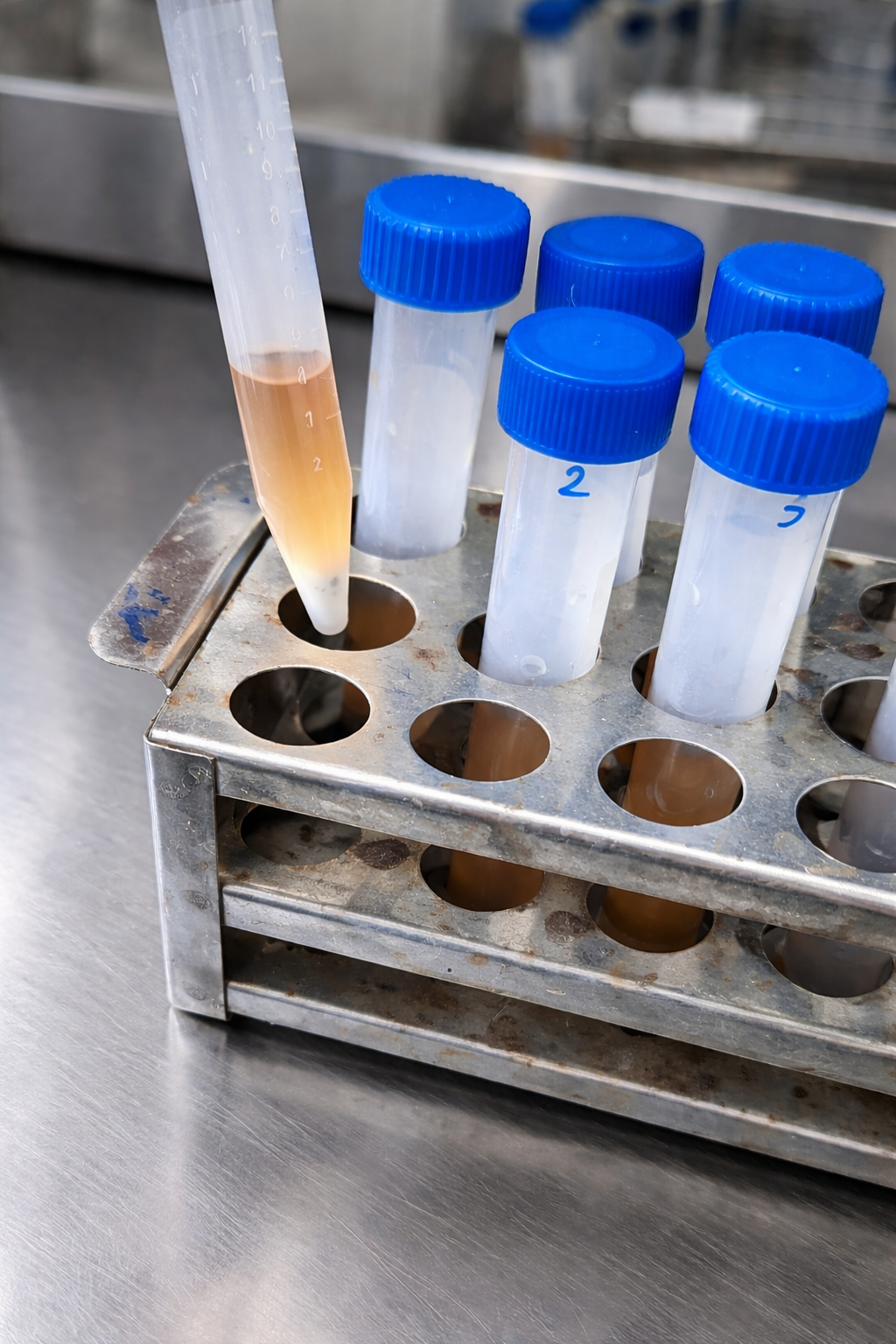

Supplement: Supplementary file 1 [file microorganisms-14-01030-s001.zip › Figure S1.png]

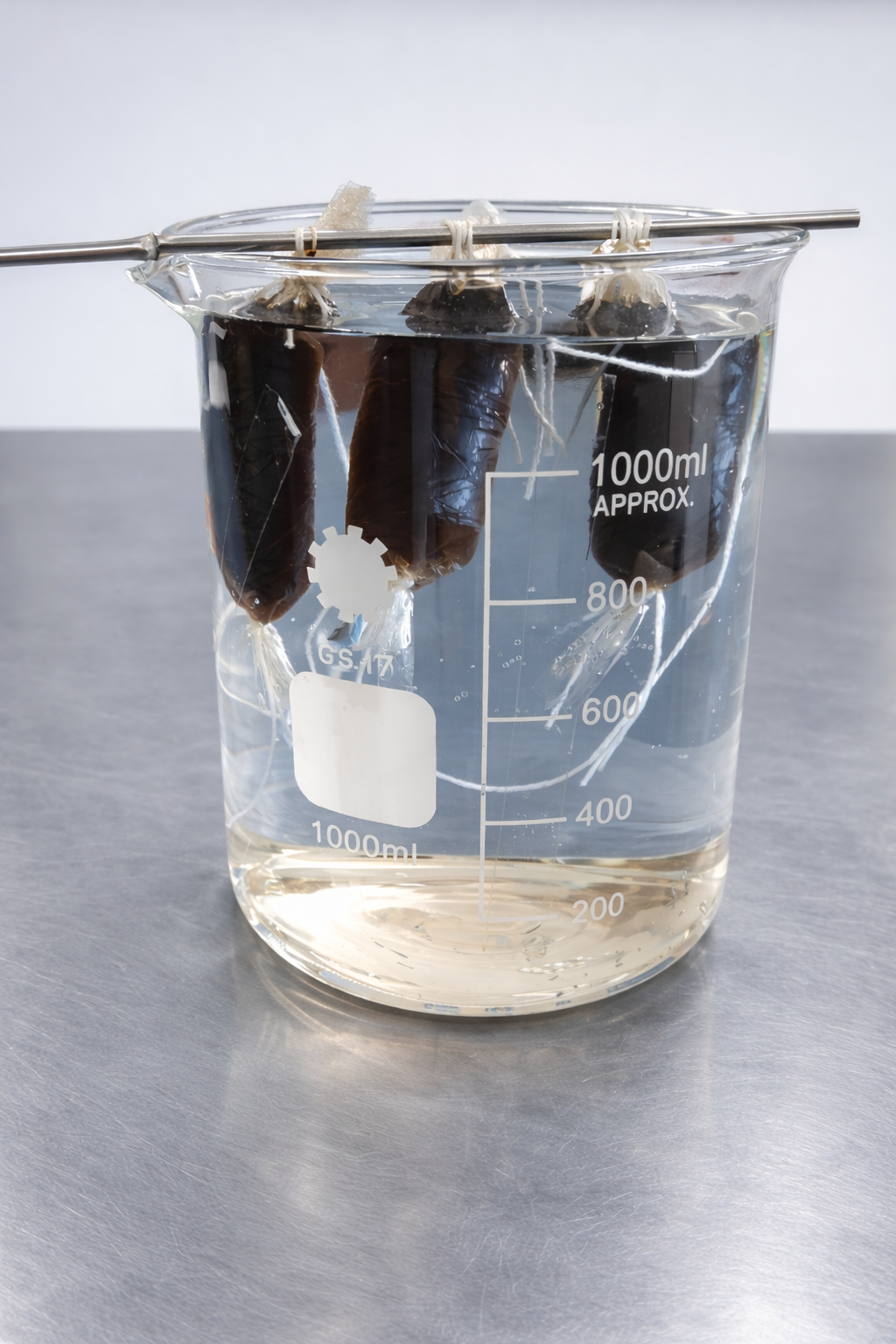

Supplement: Supplementary file 1 [file microorganisms-14-01030-s001.zip › Figure S2 - a.png]

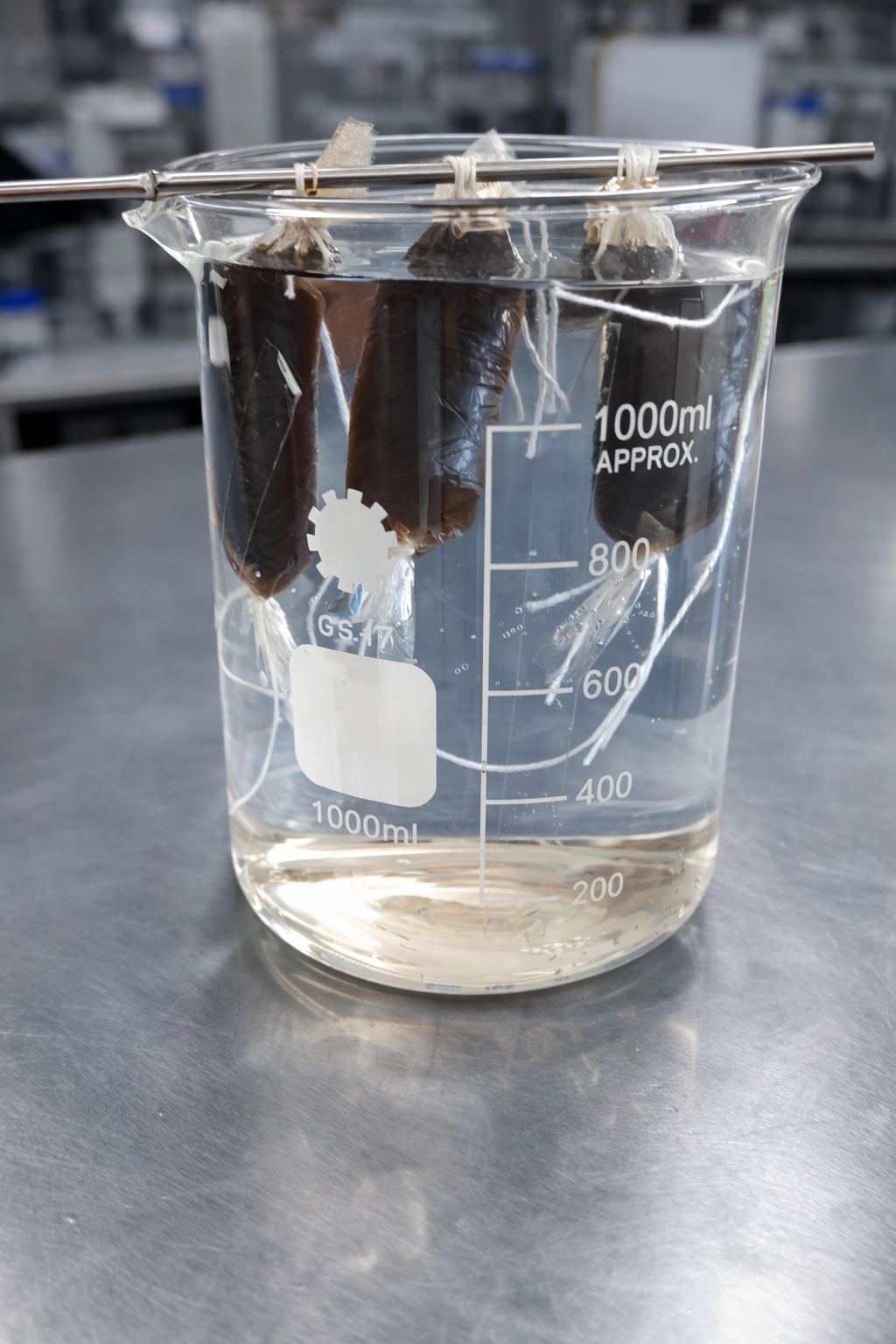

Supplement: Supplementary file 1 [file microorganisms-14-01030-s001.zip › Figure S2-b.png]
